# Comprehensive characterization of waterlogged archaeological wood by NMR relaxometry, diffusometry, micro-imaging and cryoporometry

**DOI:** 10.1039/d4cp02697g

**Published:** 2024-10-22

**Authors:** Valeria Stagno, Otto Mankinen, Sarah Mailhiot, Ville-Veikko Telkki, Silvia Capuani

**Affiliations:** a Earth Sciences Department, Sapienza University of Rome, Piazzale Aldo Moro 5 00185 Rome Italy valeria.stagno@uniroma1.it +39 0649913928; b National Research Council – Institute for Complex Systems (CNR-ISC) c/o Physics Department Sapienza University of Rome, Piazzale Aldo Moro 5 00185 Rome Italy; c NMR Research Unit, University of Oulu, Pentti Kaiteran katu 1 90014 Oulu Finland

## Abstract

Chemical, physical, and biological decay may partially or totally hide the historical and technological information carried by waterlogged wood. Investigation of the above-mentioned decay processes is essential to assess the wood preservation state, and it is important to find new methods for the consolidation and safeguarding of wooden archaeological heritage. A conventional method for assessing the wood preservation state is light microscopy. However, the method requires sample slicing, which is destructive and challenging when dealing with fragile and spongy submerged remains of heritage wood. To this end, a promising alternative non-destructive technique is proton nuclear magnetic resonance (^1^H-NMR) which considers wood as a porous system. This work aimed to perform a comprehensive analysis of structures, porosity, water distribution, decay, and possible structural inclusions of three archaeological waterlogged wood fragments of the Roman age using NMR relaxometry, micro-imaging (μ-MRI), NMR diffusometry, and NMR cryoporometry. The results were compared with a similar analysis of the three contemporary wood samples of the same species. The multimodal approach presented in this study allowed us to cover all the dimensional scales of wood, from nanometers to sub-millimeters, and reconstruct the alteration of the entire archaeological wood fragment caused by degradation.

## Abstract 2

The combination of advanced NMR techniques allows to investigate all the dimensional scale of wood (image created with BioRender).

## Graphical abstract

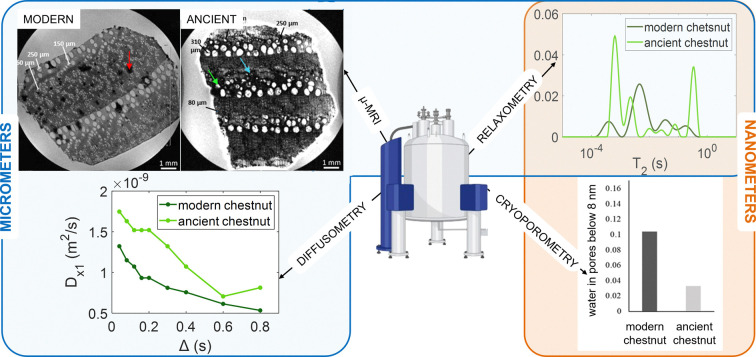

## Introduction

Archaeological wood remains are essential for understanding past human activities and raw materials technology.^1^ Under waterlogged conditions, the information carried by these remains may be partially hidden or lost due to physico-chemical and biological degradation, which induces an alteration of the wood structure depending on the properties of the wood species as well as environmental factors of the deposition site.^2–4^ Beyond physico-chemical and biological decay, tunnelling and erosion bacteria may lead to a microbial process in the presence of oxygen and anoxic conditions, respectively.^4,5^ Wood can also be attacked by soft-rot fungi when the oxygen level increases.^2,6^ Erosion bacteria produce the destruction of the cell walls and pit membranes and the filling of cell lumina with decay products from the cell wall layers.^6,7^ Along with the decay products, salts, and metals may be deposited within the wood depending on their concentration in water and the composition of the soil in which the object was buried.^8–11^ The assessment of the wood preservation state, based on the investigation of the above-mentioned degradation processes, is important for the implementation of new methods and materials to consolidate and safeguard the wooden archaeological heritage.^12,13^ For waterlogged archaeological wood, the main conservation practice implies the use of consolidating materials that penetrate in the wood microstructure and replace water.^14–19^ Sometimes, these consolidants interact with the impurities (*i.e.*, salts and metals) stored in the archaeological wood remains, compromising their conservative effect.^14^ Therefore, knowledge of porosity, water distribution, decay and possible structural inclusions in the wood is crucial for the choice of an appropriate consolidating product.

A conventional way for assessing the wood preservation state is light microscopy. This is an optical imaging technique classified as destructive, since it requires sampling a piece of wood^20^ and manually cutting it into thin sections. When dealing with the waterlogged archaeological wood, the main limitations of light microscopy are related to the sample volume^21^ and the fact that it is often used to provide qualitative images. The first limitation may be partially solved by selecting only one small sample in areas that would not affect the value of the object.^22^ However, with conventional light microscopy, even a small sample is destroyed to obtain a thin section compromising its use for further analysis. Moreover, because of the brittle and spongy texture of archaeological waterlogged wood remains, obtaining good informative images by conventional light microscopy can be a challenging task. Alternatively, reflected light microscopy and confocal laser scanning microscopy techniques^23–25^ may be used since they do not require a thin section but are based on the observation of the external surface of the wood remains. However, the water present within the sample and on its surface reflects light making the application of these techniques on the archaeological waterlogged wood very difficult.

In the field of heritage waterlogged wood conservation, recent works^20,26–33^ have shown that a promising alternative technique to light microscopy is represented by proton nuclear magnetic resonance (^1^H-NMR). It is based on the stimulation of nuclear spins by radiofrequency (RF) waves in the presence of a static magnetic field. Under these conditions, an electric signal (NMR signal) is received by the RF probe as an electromotive force. The NMR signal is proportional to the proton density and it decays exponentially in time because of transverse (spin–spin) relaxation processes caused by local oscillating fields due to molecular motion.^34^ These local oscillating fields both affect the transverse relaxation, with a time constant *T*_2_, and the longitudinal relaxation, with a time constant *T*_1_.^35–39^ However, because of the static magnetic field inhomogeneities, which contribute to the spins’ dephasing, the effective transverse relaxation time is known as *T*_2_*. Superimposing over the main static magnetic field time-dependent and controlled magnetic field gradients, NMR images and diffusion experiments are obtained.^36^ As the NMR signal depends on several parameters, such as the relaxation times *T*_1_, *T*_2_, and *T*_2_* and the diffusion coefficient *D*, it is possible to perform a multiparametric and multimodal investigation of the wood object acquiring both non-imaging and imaging experiments. MR images weighted on one of the above-mentioned parameters provide a semi-quantitative and direct observation of the wood structure and the measurement of these parameters, by means of NMR experiments based on the signal analysis, allows the quantitative and indirect inspection of the physico-chemical and physiological aspects of the wood. Importantly, NMR is a non-destructive technique that allows the preservation of the wood sample for further analyses. All the aforementioned NMR techniques have already been used to study ancient wood.^9,26,40–44^ However, magnetic resonance imaging (MRI) and diffusion NMR techniques do not allow the investigation at the sub-micrometer and nanometer scale, which is the dimensional scale characteristic of the cell wall which is the wood constituent most affected by degradation processes. With conventional diffusion techniques, the detection limit is essentially related to the diffusion length which in the Stejskal–Tanner model is set around 10 microns.^45,46^ Also, micro-imaging (μ-MRI), when performed using strong magnetic field gradients, allows a maximum linear resolution of about 10 μm.^29^ On the other hand, relaxation times may provide indirect information about the nanometer scale features but they are also sensitive to paramagnetic ions that reduce the relaxation constants and cause the appearance of unrealistic pore distributions in mathematical models used.^28,47^ In this scenario, NMR cryoporometry represents a non-destructive method to also investigate the cell wall scale and, combined with other NMR techniques, cover the entire wood dimensional scale.^44,48^

One limitation of high-field NMR and microscopic MRI is that the sample size is typically limited to a few millimeters or to a few centimeters depending on the instrument. However, surfaces of larger cultural heritage wood samples can be studied using low-field, portable single-sided NMR devices up to a couple of centimeters depth. Previous works^28,49–55^ have shown that it is possible to perform relaxometry and diffusometry experiments with a single-sided NMR instrument without sampling the wood artwork. Nevertheless, these mobile devices are characterized by highly inhomogeneous magnetic fields and are not equipped with either imaging gradients or a cooling system. Therefore, despite allowing studies on unlimited sample sizes outside a laboratory, portable NMR has lower resolution and does not enable micro-imaging and cryoporometry measurements.

In this work, three waterlogged archaeological wood samples (V century AD) were studied using a combination of advanced NMR techniques. The three ancient wood samples were identified as common spruce (*Picea abies* Karst.), sweet chestnut (*Castanea sativa* Mill.), and maple (*Acer* L.), and their preservation state was already investigated using conventional light microscopy in our previous work.^28^ The evaluation of decay was obtained by both normal and polarized light microscopy. A strong microbial decay was observed for spruce wood, which was associated with both cellulolytic bacteria and fungi. This decay resulted in a layer of dark-coloured substance, formed by erosion residual wood products, which appeared mainly in the secondary wall of latewood tracheids, facing the cell lumen. Polarized light microscopy highlighted the well-preserved structure of compound middle lamellae and secondary cell walls. Conversely, the inner part of the secondary walls was converted into an amorphous substance. A fungal attack was testified by the presence of hyphae passing through the cells and typical soft rot erosion tunnels. Moreover, inclusions were observed in both earlywood and latewood vessels of the chestnut wood and in some cases small vessels were completely obstructed. These black-coloured inclusions were attributed to iron–tannin precipitates.^8,9^ A generalized detachment and loss of fibre secondary walls was also observed in chestnut, whereas in the maple wood no complete cell wall detachment was seen. Nevertheless, the weak and spongy consistency of the maple wood indicated a significant decay, and the thinning of the fibre cell wall as well as the presence of spores and residues of fungal hyphae in the cellular lumens were observed.

This work aimed to obtain a comprehensive analysis of archaeological waterlogged wood structures, along with their porosity, water distribution, decay, and potential structural inclusions, combining four advanced NMR techniques: relaxometry, micro-imaging, diffusometry, and cryoporometry. To this end, the three archaeological wood fragments of the Roman age were compared with three contemporary wood samples of the same species. The multi-analytical approach presented in this study allowed us to cover all the dimensional scales of wood, from nanometers to millimeters, and reconstruct the state of conservation of the entire archaeological wood fragment. The results obtained from the NMR protocol presented in this work were interpreted with the support of the results obtained from decay evaluation by light microscopy carried out in our previous work.^28^

## Experimental

### Materials

Three small fragments were collected from three archaeological wood poles of a wooden pier dated to the Roman age (V century AD) and recovered in 2018 in the archaeological excavation area of the ancient harbor of Neapolis (Naples, Italy).^56,57^ Their botanical species were identified in previous works^57–62^ as common spruce (*Picea abies* Karst.), sweet chestnut (*Castanea sativa* Mill.) and maple (*Acer* L.). These wood samples were waterlogged and well preserved in the seawater of Naples (Mediterranean Sea). After the collection, the samples were kept in sealed containers full of distilled water. Moreover, three modern wood samples belonging to the same botanical species of the three ancient wood samples were used for comparison. All six wood samples (three ancient kinds of wood samples and three modern kinds of wood samples) had cylinder-like shapes of height 1 cm and diameter of less than 1 cm so that they could be inserted into the 10 mm NMR tube. Before starting the NMR measurements, the three modern wood samples were soaked with distilled water until complete saturation (corresponding to the sinking of the sample) and maintained in test tubes full of distilled water. During the relaxometry, diffusometry and cryoporometry experiments, all six samples were inserted without water in the NMR tube that was sealed with Parafilm to prevent the wood dehydration. Conversely, during μ-MRI acquisitions, the samples were inserted in the NMR tube with water and sealed with Parafilm to prevent water evaporation.

### Methods

#### Relaxometry

The spin–lattice relaxation time (*T*_1_) and the spin–spin relaxation time (*T*_2_) were measured with TopSpin 3.0 software using a Bruker-Avance III spectrometer with a ^1^H resonance frequency of 300 MHz and a 7.04 T magnetic field strength. For the *T*_1_ acquisition, the saturation-recovery (SR) sequence^63^ was used instead of the inversion-recovery (IR)^64^ to avoid the effect of radiation damping.^65^ The recovery time varied from 10 μs to 10 s in 128 points, the repetition time (TR) was 10 s, and the number of scans (NS) was 4. *T*_2_ measurements were performed with the Carr–Purcell–Meiboom–Gill (CPMG) sequence^66^ using an echo-time (TE) of 0.5 ms, and number of echoes of 10 000; all the echoes were collected in a single transient, TR = 8 s, and NS = 4.

#### Micro-imaging

High-resolution images were obtained using a Bruker-Avance 400 MHz spectrometer with a 9.4 T magnetic field. A 10 mm micro-imaging probe equipped with high-performance and high-strength magnetic field gradients was mounted and the maximum gradient strength was 1200 mT m^−1^ with a rise time of 100 μs. Three images along the three anatomical directions of the wood, which correspond to three MRI virtual sections, were acquired for each wood sample. In this way, a complete virtual histology, composed of one cross-section and two longitudinal sections, similar to that traditionally carried out by light microscopy, was obtained. The three images/virtual slices were oriented as follows: perpendicular to the wood grain (transversal section), tangent to the annual ring boundary (tangential section), and parallel to the wood ray/perpendicular to the annual ring boundary (radial section). All the MRI experiments were acquired using ParaVision 5.1 software, which allowed us to set and control the acquisition parameters, such as the number of scans (NS), number of slices (N° slices), slice thickness (STK), field of view (FOV), image matrix (MTX), in-plane resolution (R), and echo time/repetition time ratio (TE/TR). The FOV and MTX were chosen equal in both image dimensions. After some tests, the *T*_2_*-weighting (*T*_2_*-w) of images turned out the optimal solution for all the wood samples except for the ancient chestnut for which, instead, the *T*_2_-weighted (*T*_2_-w) images provided a better discrimination among different tissues and less image artifacts. The *T*_2_*-w images were acquired using either a gradient echo fast imaging (GEFI) sequence^67^ or a mic-flash sequence,^68^ whereas the *T*_2_-w images were acquired using a multi-slice multi echo (MSME) sequence.^69^ All the acquisition parameters and the sequences used were chosen to take into account the specific structural characteristics of each wood type and their degree of preservation (*e.g.*, the presence of impurities that can cause artifacts on the image), which were previously assessed in our work.^28^ The MRI protocols used for the ancient wood samples are reported in Table 1, while those used for the modern wood samples are in Table 2.

**Table 1:** Acquisition parameters used to obtain high-resolution images of ancient spruce, ancient chestnut, and ancient maple samples. *T*_2_*-w images were obtained using a GEFI sequence, whereas *T*_2_-w images using an MSME sequence

Wood sample	Ancient spruce			Ancient chestnut			Ancient maple		
Image weighting	*T*_2_*-w			*T*_2_-w			*T*_2_*-w		
Slice orientation	Transversal	Tangential	Radial	Transversal	Tangential	Radial	Transversal	Tangential	Radial
TE/TR (ms)	5.5/500	5/1500	5/1500	2.8/2000	2.6/2000	2.6/2000	5/2000	4/2000	4/2000
NS	256	128	128	256	256	256	128	128	128
No of slices	10	10	10	10	10	10	10	10	10
STK (μm)	200	300	300	250	250	250	250	250	250
FOV (cm)	0.9	0.9	0.9	0.8	1.1	1.1	0.8	1.3	1.1
MTX	1024	512	512	512	512	512	512	512	512
*R* (μm^2^)	9 × 9	18 × 18	18 × 18	16 × 16	21 × 21	21 × 21	16 × 16	25 × 25	21 × 21

**Table 2:** Acquisition parameters used to obtain high-resolution images of modern spruce, modern chestnut, and modern maple samples. Images were obtained using a GEFI sequence for spruce and a mic-flash sequence for chestnut and maple

Wood sample	Modern spruce			Modern chestnut			Modern maple		
Image weighting	*T*_2_*-w			*T*_2_*-w			*T*_2_*-w		
Slice orientation	Transversal	Tangential	Radial	Transversal	Tangential	Radial	Transversal	Tangential	Radial
TE/TR (ms)	5.1/800	4.9/800	4.9/800	2.7/1000	2.6/1000	2.6/1000	2.7/1000	2.5/1000	2.7/1000
NS	100	128	128	256	256	256	256	256	256
No of slices	4	5	5	3	3	3	3	3	3
STK (μm)	300	250	250	300	300	300	300	300	300
FOV (cm)	0.8	1.0	1.0	0.9	1.2	1.15	0.9	1.2	1.2
MTX	1024	1024	1024	512	512	512	512	512	512
*R* (μm^2^)	8 × 8	10 × 10	10 × 10	18 × 18	23 × 23	22 × 22	18 × 18	23 × 23	23 × 23

#### Diffusometry

A pulsed gradient stimulated echo (PGSTE) sequence^70^ was used to investigate the water diffusion in each wood sample with a 400 MHz spectrometer and the same probe was used for μ-MRI experiments. The PGSTE signal was obtained using a TR of 5 s, a TE of 5 ms, a diffusion gradient pulse length (*δ*) of 3 ms, and 32 steps of the gradient strength (*g*), from 26 to 1210 mT m^−1^, for each diffusion time (*Δ*). The following *Δ* values were selected according to the different wood morphologies and longitudinal relaxation times *T*_1_, so that *Δ* < *T*_1_^34,71^: 40–80–120–160–200–300–400–600–800–1000 ms. The diffusion gradient was activated along the *x* direction, *i.e.*, perpendicular to the wood grain or the vessel/tracheid main axis corresponding to the tangential direction of the wood. The *b*-value, which is defined as *b* = *y*^2^*g*^2^*δ*^2^ (*Δ* – *δ*/3), ranged from a minimum of 1.6 × 10^7^ s m^−2^ to a maximum of 9.5 × 10^11^ s m^−2^.

#### Cryoporometry

A 300 MHz Bruker-Avance III spectrometer with a 7.04 T field strength and a cooling system with a 1000 L h^−1^ airflow was used to perform the CPMG series over the temperature range of 25.4 K. The temperature was increased in 84 steps of 0.3 K from a minimum of 260.0 K to a maximum of 285.4 K. The experiment series was set using the TReNDS acquisition script^72^ in TopSpin 3.5 software. At the beginning of the temperature series, the sample temperature was left to stabilize at the lowest temperature for 2 h, then the heating rate was kept at a constant value by applying an appropriate temperature stabilization delay of 5 min K^−1^ before each experiment. The parameters in the CPMG experiments are TE = 300 μs, 1000 echoes, NS = 64, and TR = 10 s.

#### Data processing

The mean size of the characteristic porous elements of each wood type was obtained from the post-processing of the μ-MR images using ImageJ software. At least 10 counts were selected for each characteristic range of the pore size and its mean value was estimated. The diffusion coefficient (*D*) and the relaxation time *T*_1_ and *T*_2_ distributions were reconstructed in MATLAB using the inverse Laplace transform (ILT) method.^73–76^ Especially, in the case of diffusion, this approach turned out to be preferable and more robust compared to curve fitting. This is due to ILT not requiring any assumption on the number of components while it is necessary for curve fitting. As wood is a complex heterogenous system, the number of components may vary between different diffusion measurements in the same sample due to relaxation filtering effects which are pronounced when the observation time *Δ* is increased. The ILT parameters were first evaluated to find robust ones and then the same parameters were used over the whole sample set. The diffusion coefficient values were then plotted as a function of the diffusion time *Δ*.

Since wood is a complex biological system, water diffusion is restricted and shows different behaviours depending on the observation time (*Δ*). At short *Δ*, *i.e.* when 
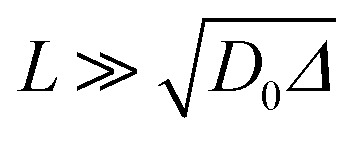
, where *L* is the length scale to probe the material structure or the typical pore size, *D*(*Δ*) depends on the surface-to-volume ratio *S*/*V*^77–81^ according to the following equation:1
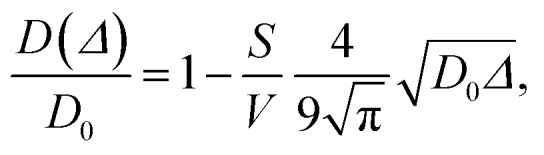
where *S* is the surface area of the pores, *V* is the volume of the pores, and *D*_0_ is the diffusion coefficient of bulk water (2.2 × 10^−9^ m^2^ s^−1^). The surface-to-volume ratio was calculated by fitting eqn (1) to the initial part of *D vs. Δ* data for all the wood samples. Then, from *S*/*V*, the pore diameter (*d*) was calculated by assuming a spherical geometry. In a totally confined geometry, the mean square displacement is bounded at long time by the size of the confining pore *L*, and as *Δ* approached infinity, *D* approaches zero.^78,81^ On the other hand, if the pores are interconnected, with long *Δ* values 
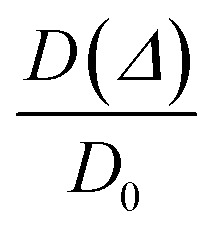
, an asymptotic value approaches

2 *D*(*Δ* → ∞)/*D*_0_ = 1/*τ*,

where *τ* is the tortuosity of pore space.^78–81^ The tortuosity is an intrinsic property of a porous material that reflects the connectivity degree of the porous network.^79,82,83^ However, the diffusion time that can be probed by NMR in a PGSTE experiment is limited by the relaxation time *T*_1_, and the asymptotic limit cannot be necessarily reached. Therefore, Padé approximation was introduced to interpolate between the short-time behaviour (eqn (1)) and the long-time asymptotic value 1/*τ*:^78,80,81,84^3
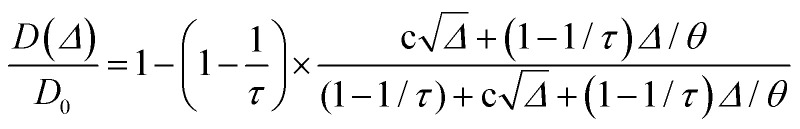
where 
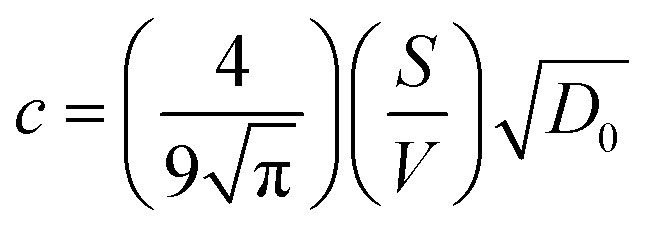
 and *θ* is a scaling constant proportional to the pore size.^78,84,85^ This approximation was used to obtain the tortuosity for chestnut and maple samples. Since several diffusion components, *D*, were measured in each wood sample, for each *D* component, a tortuosity value was computed using either eqn (2) or eqn (3) and then an average tortuosity weighted on *D* populations was obtained.

In NMR cryoporometry, the melting point of a substance confined to a small pore is lower than that of a bulk substance, according to the Gibbs–Thomson equation:^44,86^4
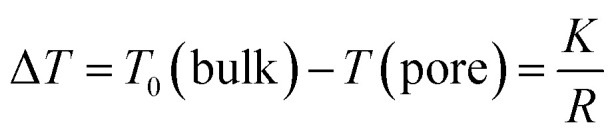
where *T*_0_(bulk) is the melting point of the bulk substance and *T*(pore) is the melting point of the substance confined in a pore with a radius *R*. *K* is a constant characteristic of the substance, in our case *K* = 30 K nm, which corresponds to the value experimentally determined for water absorbed in controlled pore glasses.^87^ The intensity of the NMR signal is proportional to the volume of the pores containing the molten liquid at a given temperature and the pores size distribution is given by the following equation:^44,88–90^5
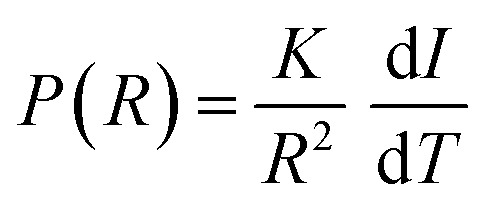
where *R* is the pore radius and d*I*/d*T* is the derivative of the NMR signal intensity *vs.* the temperature curve. However, with the experimental setup used in this study, which is intrinsically limited by instrumental characteristics, at the lowest temperature (*T* = 260 K), there is still an observable signal indicating that the smallest nanoscale pores are not frozen yet. Therefore, we cannot report reliable pore size distributions. Nevertheless, the measured CPMG experiments over the temperature range were analysed with the ILT method, so that individual water components could be separated. Indeed, in the CPMG sequence, the echo time was much longer than the *T*_2_ relaxation time of the solid wood and ice, but much shorter than *T*_2_ of bound and free water.^48^ In all cases, it was possible to distinguish the bound water signal (BW), water signal corresponding to micropore water (MP) and then with higher *T*_2_, earlywood tracheid/vessel lumens (EW) and latewood tracheid/vessel lumens (LW).^48^ According to literature, the moisture components of few millisecond relaxation times are usually associated with bound water in cell walls, and the other components with longer relaxation times, about 10–1000 ms, are associated with free water in bigger voids such as cell lumens.^91–95^ Each signal was integrated separately and plotted against temperature for further examination. Moreover, the relative amount of bound water in pores with a size of below 8 nm was calculated from the ratio of the signal intensities measured at 265 K and above the bulk melting point (273 K).^96^ This analysis is based on the fact that, according to eqn (4), only water in pores with their size of below 8 nm is unfrozen at 265 K, while above 273 K all water is unfrozen.

## Results and discussion

### Micro-imaging

Direct observation of the microstructure of our six wood samples was obtained by μ-MRI. Due to the finite echo time (2.5–5.5 ms, see Tables 1 and 2), the water components with the shortest *T*_2_ values (*T*_2_ ≤ TE) are filtered out from the images. Indeed, according to previous works,^9,10,26,27^ the contrast of μ-MR images provides important information: areas with light image voxels correspond to wood structural damages, such as holes and cavities, filled with water; areas characterized by dark image voxels indicate the accumulation of decomposed organic materials and/or heavy metals, lacking the signal due to the lack of water and fast relaxation; rounded black and large spots suggest the presence of gas bubbles due to the gas emissions of infesting microorganisms (*e.g.*, fungi). The wood morphology is discussed separately for spruce, chestnut, and maple in the following sections.

### Spruce

**Fig. 1:**
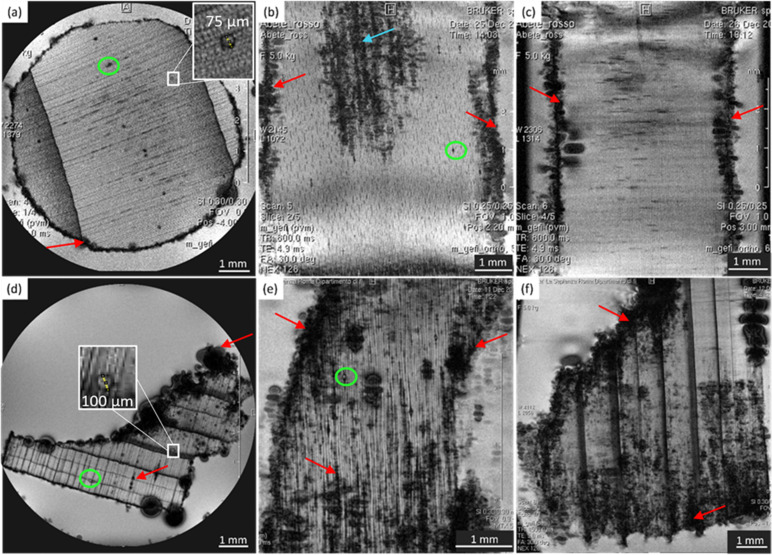
Complete virtual histology of the modern spruce (a)–(c) and the ancient spruce (d)–(f) obtained by MRI: (a) and (d) the transversal sections, (b) and (e) the tangential sections and (c) and (f) the radial sections. Green circles indicate the resin channels; red arrows indicate the black spots and image artifacts; light blue arrow points to a region with incomplete water saturation.

In Fig. 1, microscopic MR images of both the modern and the ancient spruce are shown, revealing their anatomies. The upper row (a, b, and c) of Fig. 1 displays images of the modern spruce, while the lower row (d, e, and f) shows images of the ancient spruce. In Fig. 1b, the presence of a black area (light blue arrow) in the upper part of the sample is due to the incomplete water filling of the wood structure, but it is not attributable to any decay process since it disappeared during the acquisition while the sample remained immersed in water in the NMR tube. Conversely, in all the MR images of the modern spruce, many black spots (red arrows), associated with a low water content and short *T*_2_, are seen along the sample border, which are due to both the difference in the magnetic susceptibility and the presence of infest microorganisms. From the comparison among the MR images of the modern and the ancient spruce, the first thing to notice is the presence of many more artifacts and black spots (red arrows) in the ancient wood images. These are due to strongly degraded areas with bacteria, fungi, and paramagnetic inclusions.^9,28^ Because most of the artifacts and black spots are located along the rays and on the sample border (red arrows), as visible in the transversal and tangential sections (Fig. 1d and e), we can conclude that these structures were heavily degraded. In these areas, characterized by dark voxels, the NMR signal is weak because of the lack of water or the very short *T*_2_. Another notable difference is seen in the resin channels (green circles): those of the modern spruce appear with black image voxels and short *T*_2_, whereas those of the ancient spruce are characterized by brighter voxels and long *T*_2_. This indicates that resin was lost from the resin channels of the archaeological wood sample and replaced by water. Moreover, on average, the measured resin channels in the modern spruce have a diameter of around 75 μm and in the ancient spruce around 100 μm.

### Chestnut

**Fig. 2:**
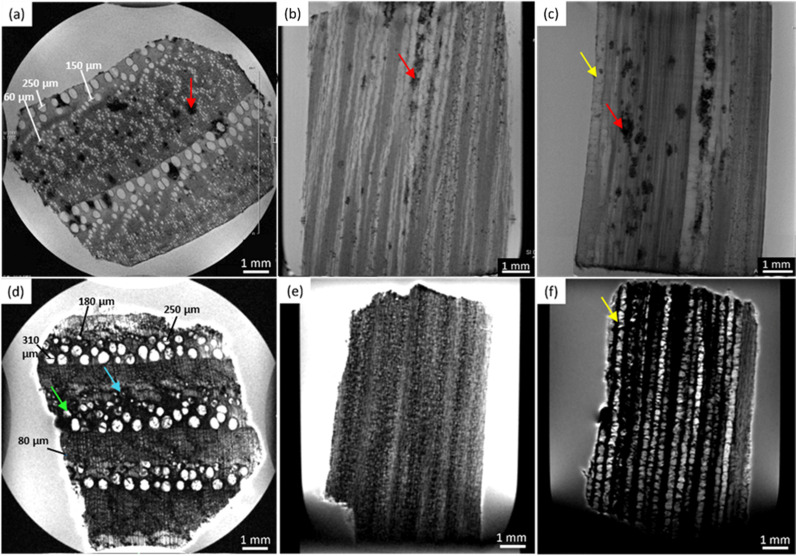
Complete virtual histology of the modern chestnut (a)–(c) and the ancient chestnut (d)–(f) obtained by MRI: (a) and (d) the transversal sections, (b) and (e) the tangential sections and (c) and (f) the radial sections. Red arrows indicate the extractive accumulations; yellow arrows indicate the vessels length with or without inclusions; green arrow points to a partially obstructed vessel and light blue arrow to a totally obstructed vessel.

Fig. 2 shows the MRI-based virtual histology of both modern and ancient chestnuts. The modern chestnut images are displayed in the upper row (a)–(c), while the ancient chestnut images are in the lower row (d)–(f). The MR sections acquired on the modern chestnut are characterized by a marked difference in the image contrast if compared with those acquired on the ancient chestnut. The archaeological chestnut wood exhibits a very dark contrast throughout the MR images, which hides its typical porous-ring structure^60^ and some regions appear with rather dark voxels associated with a low water content and short *T*_2_. Indeed, only large pores characterized by bright voxels and long *T*_2_ are fully resolved and they can be identified as vessels of the earlywood, while smaller vessels of the latewood, characterized by less bright voxels and shorter *T*_2_, are only partially visible. Conversely, in the modern chestnut image, the porous-ring structure is clearly visible and it is characterized by pores with voxels of different brightness associated with different *T*_2_ values, corresponding to vessels of different sizes, and a background with darker voxels and shorter *T*_2_, associated with parenchyma, rays and fibres. Three and four main sizes of vessels can be measured, respectively, on the transversal section of the modern and ancient chestnuts, as highlighted in Fig. 2a and d. On average, the vessel sizes, when measured along the tangential direction, are around 295 ± 31, 139 ± 33 and 60 ± 16 μm for the modern chestnut and around 355 ± 45, 263 ± 10, 170 ± 22 and 83 ± 19 μm for the ancient chestnut. Furthermore, it is worth noting that modern chestnut vessels have an elongated shape characterized by a different diameter if measured along the tangential or the radial direction; instead, the ancient chestnut vessels exhibit a more circular shape. Transversal, tangential, and radial sections of the modern chestnut (Fig. 2a–c) show the presence of regions with black voxels and very short *T*_2_ (red arrows) corresponding to the accumulation of extractives in which this wood type is naturally rich. Vessels, instead, appear almost totally free of inclusions in the modern chestnut and partially (green arrow) or totally (light blue arrow) obstructed in the ancient chestnut. The strong change in the image contrast between the modern and the ancient wood samples suggests a heavy degradation of the archaeological sample with the accumulation of inclusions and iron-impurities^9^ both in the parenchyma and in the vessel lumens, as already highlighted in our previous work.^28^ Moreover, the presence of inclusions can also be seen along the vessels in the radial section of the ancient chestnut (the yellow arrow in Fig. 2f) which, in contrast, are not present along vessels of the modern chestnut (the yellow arrow in Fig. 2c).

### Maple

**Fig. 3:**
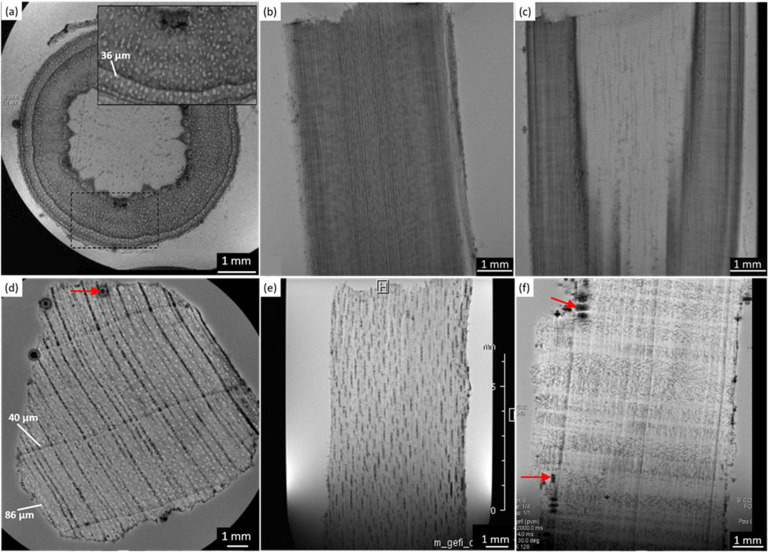
Complete virtual histology of the modern maple (a)–(c) and ancient maple (d)–(f) obtained by MRI: (a) and (d) the transversal sections, (b) and (e) the tangential sections and (c) and (f) the radial sections. Red arrows indicate image artifacts associated with the presence of decay phenomena. The modern maple sample was taken from a branch.

In Fig. 3, the MRI-reflected morphology of the modern and the ancient maple wood samples can be observed. The modern maple images are displayed in the upper row (a)–(c), while the ancient maple images are in the lower row (d)–(f). According to our previous work,^28^ in which the assessment of the conservation state of these three archaeological wood samples was obtained by light microscopy, the ancient maple wood is better preserved than the ancient spruce and chestnut samples. Indeed, only a few black artifacts can be seen on the MR images (red arrows). For comparison, we used a piece of modern maple coming from a branch of the maple tree that for this reason has a different morphology in the center of the wood piece. However, the outermost zone well represents the typical diffuse porous-ring structure of the maple characterized by vessels with bright voxels and long *T*_2_ and a background with darker voxels and shorter *T*_2_ associated with parenchyma, rays and fibers. On average, vessels measured on the modern maple have a diameter of 36 ± 8 μm, whereas vessels of the ancient maple are characterized by two different sizes of around 86 ± 10 μm and 40 ± 4 μm. In the ancient maple, the red arrows indicate the image artifacts that are usually produced by the presence of infest microorganisms.^6,9,28,47^ These artifacts were not noticed in the modern maple images.

### Relaxometry

**Fig. 4:**
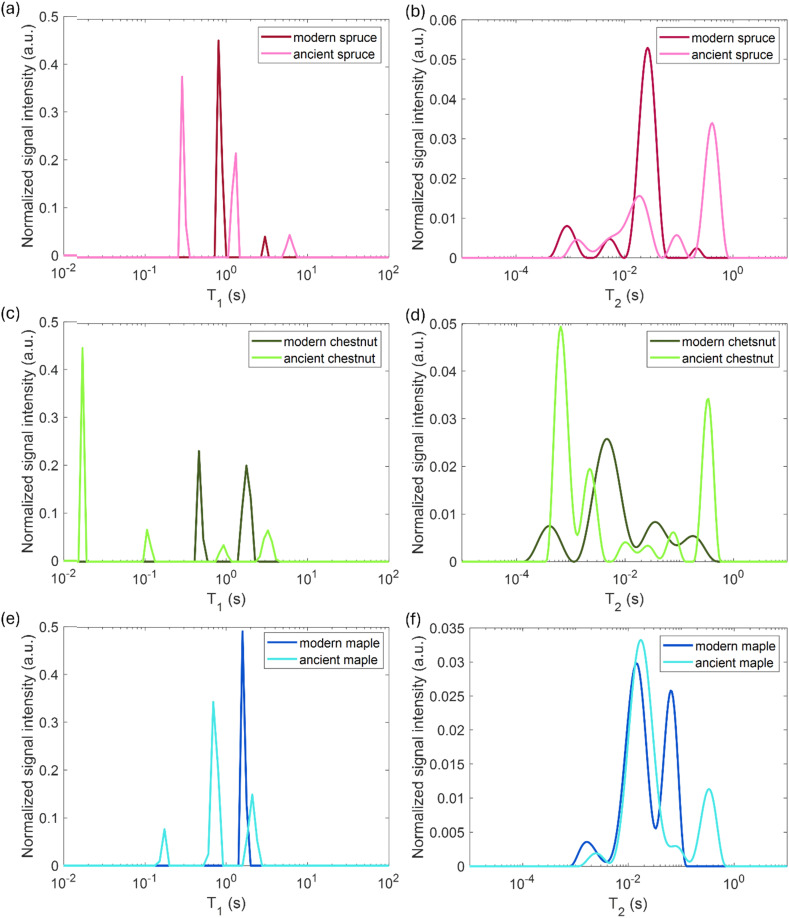
(a), (c) and (e) Spin–lattice relaxation time (*T*_1_) and (b), (d) and (f) spin–spin relaxation time (*T*_2_) distributions of modern and ancient spruce (a) and (b), chestnut (c) and (d) and maple (e) and (f) samples.

The results obtained from the relaxometry analysis are discussed separately for spruce, chestnut, and maple in the following sections, where the *T*_1_ and *T*_2_ peaks shown in Fig. 4 have been interpreted and assigned to different water populations associated with characteristic wood structures. Peak assignments to wood anatomical elements are summarized in Table 3. On average, the ancient wood samples include more relaxation components than their modern counterparts.

**Table 3:** *T*_1_ and *T*_2_ peaks assignment for the six wood samples along with their probabilities *P*_1_ and *P*_2_

Wood specimen	*T*_1_ (ms)	*P*_1_ (%)	*T*_2_ (ms)	*P*_2_ (%)	Assignment
Modern spruce	3000	9	200	4	Water on the sample surface
	800	91	27	78	EW tracheids
			5	7	LW tracheids
			0.9	12	Cell walls
Ancient spruce	6000	7	—	—	Water on the sample surface
	1300	34	400	52	Large voids due to wood decay/empty resin channels
	300	59	97	9	Large EW tracheids
			18	24	EW tracheids
			5	8	LW tracheids
			1.4	7	Cell walls
Modern chestnut	1800	46	175	11	Vessels ∼ 140–300 μm
			33	18	Vessels ∼ 60 μm
	500	54	5	55	Fibres, parenchyma
			0.4	16	Cell walls
Ancient chestnut	3000	11	350	29	Vessels ∼ 260–350 μm
	900	6	78	5	Vessels ∼ 170 μm
			26	3	
	100	11	10	3	Vessels ∼ 80 μm
	20	73	2	17	Cell walls
			0.6	42	Fibres, parenchyma
Modern maple	1600	100	70	44	Vessels ∼ 36 μm
			15	50	Fibres, parenchyma
			1.5	6	Cell walls
Ancient maple	2100	26	350	23	Vessels ∼ 86 μm
	700	60	78	6	Vessels ∼ 40 μm
			16	67	Fibres, parenchyma
	200	13	2.3	4	Cell walls

### Spruce

In Fig. 4a, the modern spruce has only two *T*_1_ components and the ancient spruce presents three different *T*_1_ components where one of the components has significantly shorter *T*_1_ than the components of the modern spruce sample. Both the modern and ancient spruce samples show a small (7–9% of the total intensity) peak at very long *T*_1_ = 3 and 6 s, respectively. These *T*_1_ peaks, along with the weak *T*_2_ component around 200 ms observed only in the modern spruce, can be attributed to excess water stored in the NMR tube outside the sample^97,98^ and they can be excluded from our anatomical discussions. In the modern spruce, the individual *T*_1_ component around 800 ms is a mean value over the entire wood structure and it is associated with the three different *T*_2_ components around 30, 5 and 0.9 ms (Fig. 4b). The most intense *T*_2_ peak is around 30 ms (78%) and it can be attributed to free water in earlywood (EW) tracheids with a larger lumen size, in good agreement with previous works.^28,44,91,98^ The small (7%) *T*_2_ peak at around 5 ms is associated with free water in latewood (LW) tracheids, which are characterized by a smaller lumen size, in agreement with the literature.^28,44,91,98^ The shortest *T*_2_, instead, can be attributed to bound water in cell walls, which represent the 12% of the total water signal. The observation of three *T*_2_ components and only one *T*_1_ component indicates that exchange of water between the three sites (bound water as well as EW and LW free water) is fast in the *T*_1_ time scale (*τ*_ex_ ≪ 800 ms) but slow in the *T*_2_ time scale (*τ*_ex_ ≥ 1–30 ms). For the ancient spruce, two different *T*_1_ peaks at around 1300 and 300 ms and five different *T*_2_ peaks at around 400, 97, 18, 5 and 1.4 ms were observed. The *T*_1_ component of 1300 ms (34%) can be associated with the *T*_2_ component of 400 ms (52%), which can be attributed to water in large voids due to wood decay and empty resin channels (*i.e.*, full of water instead of resin), with a size of around 100 μm (as measured in the MR image of Fig. 1d). The shortest *T*_1_ peak at around 300 ms, which is also the most intense (59%), can be associated with the four shorter *T*_2_ peaks at around 97, 18, 5 and 1.4 ms. It should be noted that the peak at around 5 ms is not well-resolved and it is intermediate between the above-mentioned *T*_2_ peaks at 18 and 1.4 ms, and this suggests a continuous distribution among these three different water populations. *T*_2_ = 97 ms can be ascribed to water in large EW tracheid lumen (9%), *T*_2_ = 18 ms can be ascribed to water in the rest of EW tracheid lumen (24%) and *T*_2_ = 5 ms can be ascribed to LW tracheids (8%). Finally, *T*_2_ = 1.4 ms arises from bound water in cell walls (7%). The free and bound water peaks are closer to each other than in the case of modern spruce due to the partial decay of the cell wall structure as well as related accelerated exchange between the two sites. In conclusion, both for the modern and the ancient wood samples, earlywood tracheids occupy a greater volume than latewood tracheids. The *T*_2_ of the bound water in the ancient spruce wood is significantly longer than that in the modern spruce wood and there is a continuous distribution among the bound water, earlywood tracheids water and latewood tracheid water populations. This result denotes non-isolated water compartments and confirms the increase of the permeability of the cell wall due to its degradation,^13,99^ as shown by the stretching of its *T*_2_ component.

### Chestnut

In Fig. 4c, the modern chestnut has only two *T*_1_ components, while the ancient chestnut is characterized by four *T*_1_ components. In Fig. 4d, the modern and the ancient chestnuts exhibit similar *T*_2_ values. The ancient chestnut shows two high peaks at very long and very short *T*_2_ values, and a greater compartmentalization of water *T*_2_ populations. Conversely, the modern chestnut has a major peak at an intermediate *T*_2_ value and fewer water populations. The modern chestnut sample shows two *T*_1_ components around 1800 and 500 ms, whereas it is characterized by four *T*_2_ components around 175, 30, 5, and 0.4 ms. The *T*_1_ = 1800 ms peak (46%) can be associated with *T*_2_ = 175 and 30 ms peaks, which can be attributed to 11% of water in large vessels with a size of around 140–300 μm and to 18% of water in smaller pores with a size of around 60 μm, respectively. This agrees with the pore size measured on the respective MR image in Fig. 2a. The *T*_1_ = 500 ms peak (54%), instead, can be associated with two *T*_2_ components, 5 and 0.4 ms, that can be attributed, respectively, to water in fibres and parenchyma (55%), and to water in cell walls (16%). The three *T*_2_ peaks associated with two sizes of vessels (around 140–300 and 60 μm) and with fibres and parenchyma show a continuous distribution, whereas the *T*_2_ peak associated with cell walls is separated. The most intense *T*_2_ peak is that associated with fibres and parenchyma (55%). The archaeological chestnut shows a greater compartmentalization of both *T*_1_ and *T*_2_ with four *T*_1_ components around 3000, 900, 100 and 20 ms and six *T*_2_ components of about 350, 78, 26, 10, 2 and 0.6 ms. *T*_1_ = 3000 ms (11%) and *T*_2_ = 350 ms (29%) can be interpreted as free water in very large vessels with a size of around 260–350 μm, being in good agreement with the fact that vessels with a size of around 260–350 μm are also the most abundant in the MR image of Fig. 2b. The second *T*_1_ around 900 ms (6%) is associated with two different *T*_2_ = 78 and 26 ms (5 and 3%) and these can be attributed to water in vessels with a size of 170 μm. The third *T*_1_ peak at 100 ms (11%) is associated with the *T*_2_ peak at around 10 ms (3%) and they can be attributed to water in vessels with a size of 80 μm. Moreover, there is a continuous distribution among the peaks with *T*_2_ = 78, 26 and 10 ms that indicates the existence of not discrete relaxation populations in vessels. The last peak at very short *T*_1_ of 20 ms (73%) can be associated with the two shortest *T*_2_ peaks, at 2 and 0.6 ms (17 and 42%), and they are interpreted to arise from cell walls and fibers/parenchyma, respectively. Furthermore, the continuous distribution between the water population in fibres/parenchyma and that in cell walls suggests that there is water exchange between them. In conclusion, on average, the ancient chestnut shows a greater water compartmentalization and it is characterized by longer *T*_2_ relaxation times compared to the modern chestnut but it has two *T*_1_ components that are longer than those of the modern chestnut and two *T*_1_ components that are shorter. The shortening of these two *T*_1_ components is caused by the presence of paramagnetic inclusions, *i.e.*, iron-tanning substances,^28,99^ in fibres, parenchyma, and cell walls. A strong degradation process of the wood cell wall, which has increased its water permeability and porosity, was observed, in good agreement with our previous work.^28^

### Maple

In Fig. 4e, the modern maple is characterized by only one *T*_1_ peak, whereas the ancient maple is characterized by three *T*_1_ peaks. In Fig. 4f, the modern and the ancient maple samples have a quite similar *T*_2_ distribution. However, the ancient maple shows an additional *T*_2_ component on the long side. The only one *T*_1_ component of the modern maple, around 1600 ms, can be considered a mean value over the overall structure. This *T*_1_ is associated with three different *T*_2_ at around 70, 15, and 1.5 ms that can be attributed, respectively, to water in vessels (44%) with a mean size of 36 ± 8 μm (measured on the MR image of Fig. 3a), water in fibres and parenchyma (50%), and water in cell walls (6%). There is a marked continuous distribution between the *T*_2_ peaks of vessels and fibres and the most intense peak is associated with fibres, which represent the 50% of the wood. For the ancient maple sample, the *T*_1_ component around 2100 ms and the *T*_2_ component around 350 ms indicate water in larger vessels (around 86 ± 10 μm, measured on the MR image in Fig. 3d).^97,98^ The second *T*_1_ = 700 ms can be associated with the two *T*_2_ = 78 and 16 ms and they are the characteristic, respectively, of water in smaller vessels (around 40 ± 4 μm, measured on the MR image in Fig. 3d) and water in fibres and parenchyma (67%), respectively. Here, a continuous distribution among vessels, fibres, and parenchyma can be observed. The last *T*_1_ = 200 ms is associated with the last *T*_2_ = 2.3 ms and they can be attributed to cell walls (4%). For the modern sample, also in the ancient sample, fibres are the most abundant element and constitute 67% of the wood.

In conclusion, there is a greater compartmentalization of *T*_1_ in the ancient maple compared to the modern one. This may indicate possible structural changes in the ancient maple induced by a decay process. These changes are confirmed by the increase of *T*_2_ associated with the cell walls and the larger size of vessels in the ancient sample compared to the modern sample. However, it is worth noting that, while the specimen comes from the tree trunk for the ancient maple, the specimen was collected from a branch for the modern maple and this may be the reason of some structural differences observed in the two specimens.

### Diffusion

**Fig. 5:**
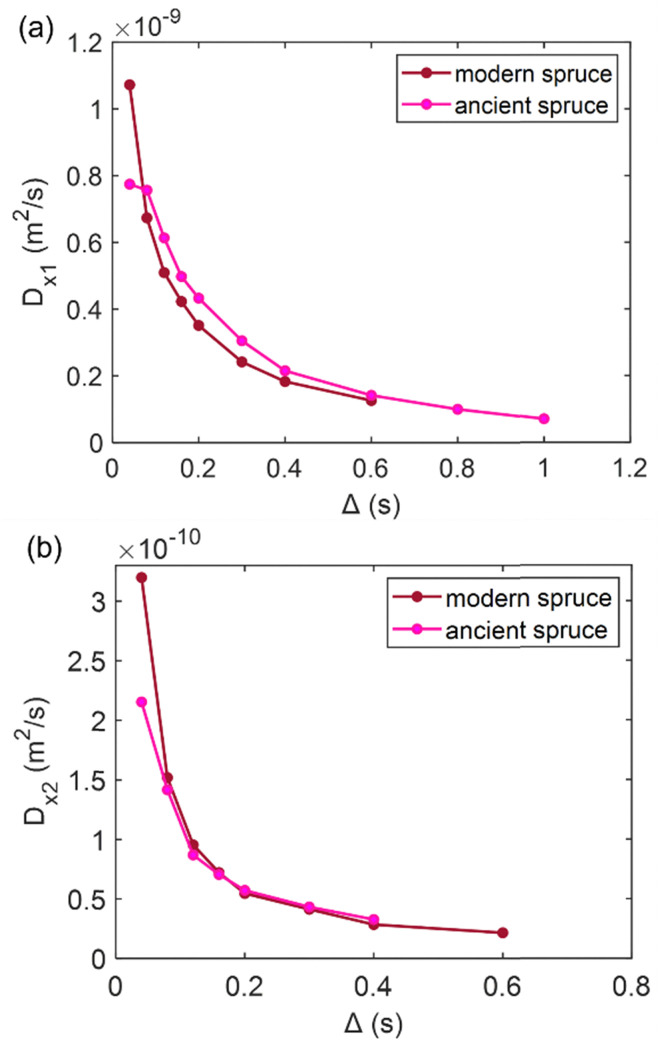
(a) The first component (*D*_*x*1_) and (b) the second component (*D*_*x*2_) of the diffusion coefficient (*D*) measured along the *x*-axis (perpendicular to the wood grain) as a function of the observation time (*Δ*) and calculated using the ILT method^76^ for modern and ancient spruce samples. The intensity ratio between *D*_*x*1_ and *D*_*x*2_ components is 1 : 0.10 and 1 : 0.26 for the modern spruce and the ancient spruce, respectively.

**Fig. 6:**
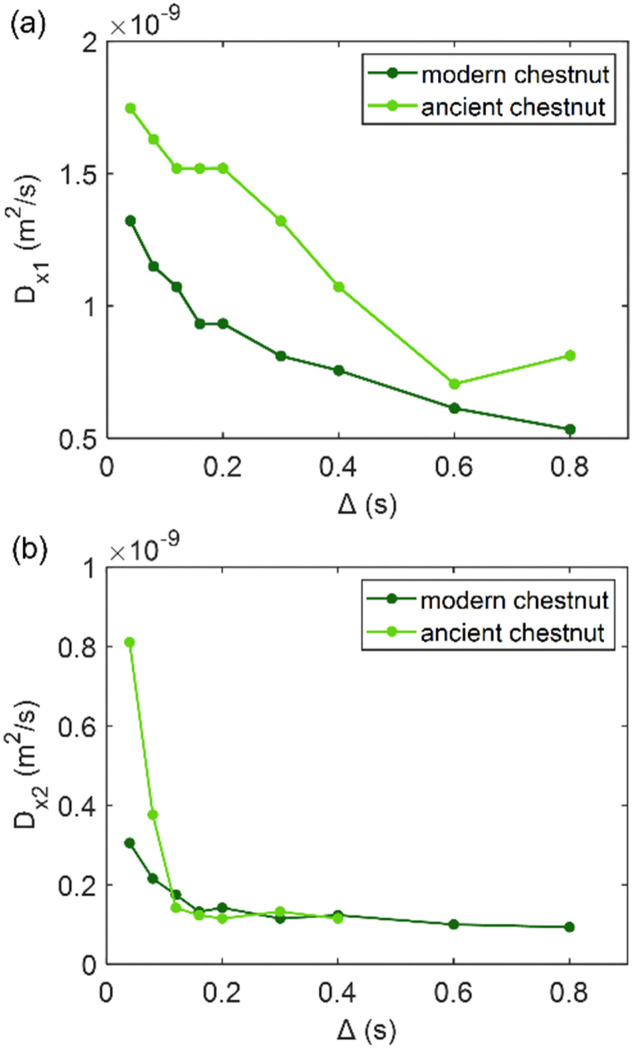
(a) The first component (*D*_*x*1_) and (b) the second component (*D*_*x*2_) of the diffusion coefficient (*D*) measured along the *x*-axis (perpendicular to the wood grain) as a function of the observation time (*Δ*) and calculated using the ILT method^76^ for modern and ancient chestnut samples. The intensity ration ratio between *D*_*x*1_ and *D*_*x*2_ components is 1 : 0.30 and 1 : 0.47 for the modern chestnut and the ancient chestnut, respectively.

**Fig. 7:**
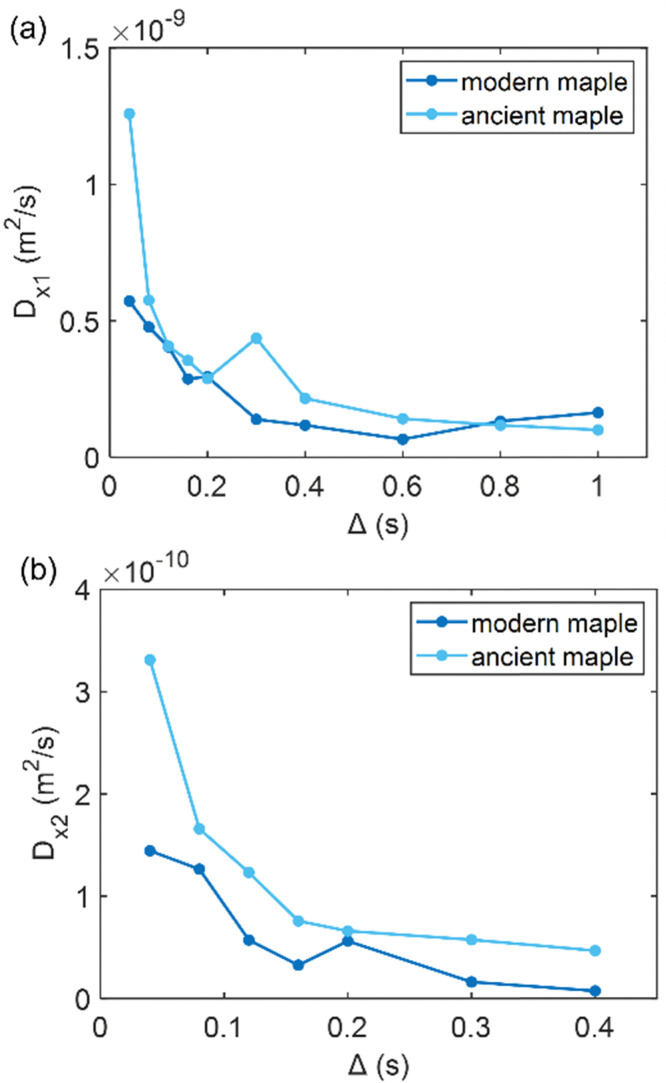
(a) The first component (*D*_*x*1_) and (b) the second component (*D*_*x*2_) of the diffusion coefficient (*D*) measured along the *x*-axis (perpendicular to the wood grain) as function of the observation time (*Δ*) and calculated using the ILT method^76^ for modern and ancient maple samples. The intensity ratio between *D*_*x*1_ and *D*_*x*2_ components is 1 : 0.38 and 1 : 0.06 for the modern maple and the ancient maple, respectively.

Diffusion of water in the wood samples was measured in the tangential direction. Because in the diffusion experiments, magnetization remained in the transverse plane altogether for 10 ms before detection, and the bound water signal was filtered out due to its short *T*_2_. Altogether, three diffusion decay components were observed for all samples. However, for one of the components, the observed apparent diffusion coefficient value did not change when the observation time was increased, indicating non-restricted diffusion. Therefore, this component was interpreted to arise from bulk water on sample surfaces. For the two other diffusion components, the apparent diffusion coefficient values (*D*) as a function of the observation time (*Δ*) obtained from ILT analysis are shown in Fig. 5–7. The diffusion coefficients decrease with the increasing observation time due to restricted diffusion. The resulting surface-to-volume ratio (S/V) obtained from eqn (1) and the pore diameter (*d*), calculated from *S*/*V*, with the ratio between the weights (*p*_1_ : *p*_2_) of the two *d* components are reported in Table 4, along with the average tortuosity (*τ*) obtained respectively from eqn (2) for spruce samples and from eqn (3) for chestnut and maple samples.

**Table 4:** Surface-to-volume ratio (*S*/*V*) obtained from eqn (1), pore diameter (*d*) calculated from *S*/*V* together with the ratio between the weights of the two *d* components (*p*_1_ : *p*_2_), and mean tortuosity (*τ*) calculated as the weighted average between the tortuosity obtained for the two diffusion populations from eqn (2) for spruce samples and from eqn (3) for chestnut and maple samples

	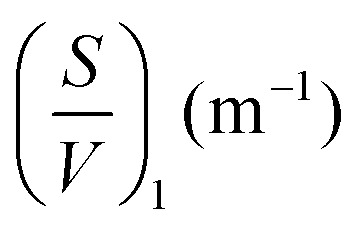	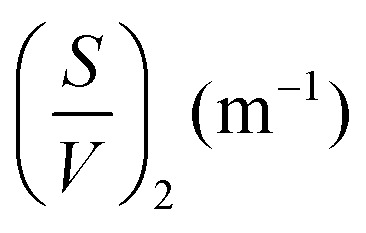	*d*_1_ (μm)	*d*_2_ (μm)	*p*_1_ : *p*_2_	*τ*
Modern spruce	(1.9 ± 0.1) × 10^5^	(2.5 ± 0.3) × 10^5^	31.7 ± 1.6	24.4 ± 2.8	1 : 0.10	16.9 ± 1.1
Ancient spruce	(1.7 ± 0.1) × 10^5^	(2.5 ± 0.3) × 10^5^	35.7 ± 1.8	24.2 ± 2.9	1 : 0.26	23.3 ± 1.3
Modern chestnut	(1.3 ± 0.1) × 10^5^	(2.4 ± 0.3) × 10^5^	45.2 ± 2.9	25.1 ± 3.0	1 : 0.30	7.2 ± 2.0
Ancient chestnut	(7.4 ± 0.4) × 10^4^	(2.3 ± 0.1) × 10^5^	81.5 ± 4.5	26.5 ± 1.6	1 : 0.47	23.9 ± 0.2
Modern maple	(2.1 ± 0.2) × 10^5^	(2.5 ± 0.3) × 10^5^	28.1 ± 2.9	23.7 ± 3.0	1 : 0.38	65.2 ± 0.7
Ancient maple	(1.9 ± 0.1) × 10^5^	(2.4 ± 0.3) × 10^5^	30.8 ± 1.5	24.6 ± 2.8	1 : 0.06	87.5 ± 0.6

It is worth noting that the major weakness of NMR diffusion is that the maximum length scale covered by this technique (on the order of 
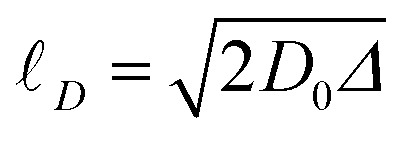
, where *D*_0_ = 2.2 × 10^−9^ m^2^ s^−1^ is the bulk water diffusion coefficient) is intrinsically limited by the characteristic *T*_1_ of the wood material, which limits the maximum diffusion delay (*Δ* < *T*_1_).

The results obtained from diffusion analyses are discussed separately for spruce, chestnut and maple in the following sections.

### Spruce

In Fig. 5, the water diffusion measured perpendicular to the wood grain is quite similar both in the modern and in the ancient spruce samples. The two diffusion components, *D*_*x*1_ and *D*_*x*2_, are associated with water inside pores of sizes *d*_1_ = 31.7 ± 1.6 μm and *d*_2_ = 24.4 ± 2.8 μm in the modern spruce and with pores of sizes *d*_1_ = 35.7 ± 1.8 μm and *d*_2_ = 24.2 ± 2.9 μm in the ancient spruce, respectively. The pore size *d*_1_ can be attributed to the lumen size of earlywood tracheids, whereas the pore size *d*_2_ can be attributed to the lumen size of latewood tracheids, in good agreement with results reported in the literature.^100^ Both *D*_*x*1_ and *d*_1_ are a bit higher in the ancient spruce indicating that their earlywood tracheids have a bit larger lumen size than those of the modern spruce. The relative intensity of the earlywood component was about 10 times higher than that of the latewood component, being in good agreement with the relaxation experiments. The mean tortuosity is higher in the ancient spruce (*τ* = 23.3 ± 1.3) than in the modern spruce (*τ* = 16.9 ± 1.1). This result suggests that on average water motion in the archaeological sample is more tangled than in the modern sample.

### Chestnut

Fig. 6 shows that on average the first component *D*_*x*1_ of the diffusion coefficient in the archaeological chestnut is higher than that in the modern chestnut. Conversely, the component *D*_*x*2_ is similar in both samples. The two derived pore sizes *d*_1_ and *d*_2_ can be associated with small vessels and with fibers and parenchyma, respectively. For the ancient chestnut, *d*_1_ = 81.5 ± 4.5 μm, whereas for the modern chestnut *d*_1_ = 45.2 ± 2.9 μm. This result indicates that vessels are smaller in the modern chestnut compared to the ancient one. Indeed, this pore size can be attributed to the smallest size of vessels that were measured on the μ-MR image for both wood samples (Fig. 2a and d). μ-MRI showed that the smallest size of vessels in the ancient chestnut (∼80 μm) was larger than that in the modern chestnut (∼60 μm). The pore size *d*_2_ = 26.5 ± 1.6 for the ancient chestnut and *d*_2_ = 25.1 ± 3.0 for the modern chestnut suggests that fibres and parenchyma have a similar size in both wood samples. The mean tortuosity is three times greater in the archaeological sample (*τ* = 23.9 ± 0.2) than that in the modern sample (*τ* = 7.2 ± 2.0) indicating that water motion is less easy in the archaeological sample. This result can be explained considering the high content of inclusions (*i.e.* iron-tanning substances^28,99^) stored in the structure of the ancient wood, as observed in the MR images (Fig. 2d–f). The inclusions lead to the formation of new compartments and water pools that make the ancient wood structure more complex.

### Maple

Fig. 7 shows that the two diffusion components, *D*_*x*1_ and *D*_*x*2_, are quite similar in the modern maple and ancient maple. These two water diffusion components are associated with two different pore sizes, *d*_1_ and *d*_2_, that are = 28.1 ± 2.9 and 23.7 ± 3.0 μm for the modern maple and 30.8 ± 1.5 and 24.6 ± 2.8 μm for the ancient maple, respectively. The intrinsic limitation due to the *T*_1_ only allowed us to probe water diffusion in small vessels (quantified by the diameter *d*_1_) of the ancient maple but not in the large vessels (with a size of around 86 μm) measured instead on the MR image (Fig. 3d). From diffusion, both the vessels size (*d*_1_) and the size of fibres and parenchyma (*d*_2_) are quite similar for both the ancient and modern samples. Moreover, it seems that vessels and fibers have a similar size in the modern sample. However, the mean tortuosity is greater in the ancient maple (*τ* = 87.5 ± 0.6) than that in the modern one (*τ* = 65.2 ± 0.7). This result suggests that on average water moves hardly in the archaeological wood.

### Cryoporometry

**Fig. 8:**
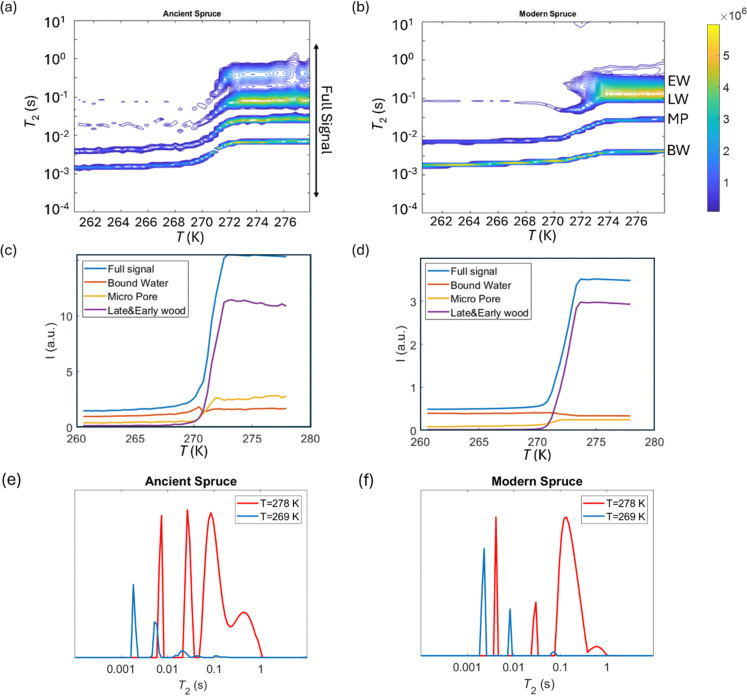
*T*_2_ relaxation distributions *versus* temperature for ancient spruce (a) and modern spruce (b). EW, LW, MP, and BW refer to earlywood, latewood, micropore and bound water signals, respectively. The signal intensity of each component *versus* temperature for ancient spruce (c) and modern spruce (d) and the full signal region indicated in (a). *T*_2_ distributions for ancient spruce (e) and modern spruce (f) before and after the melting point (*T* = 273 K).

**Fig. 9:**
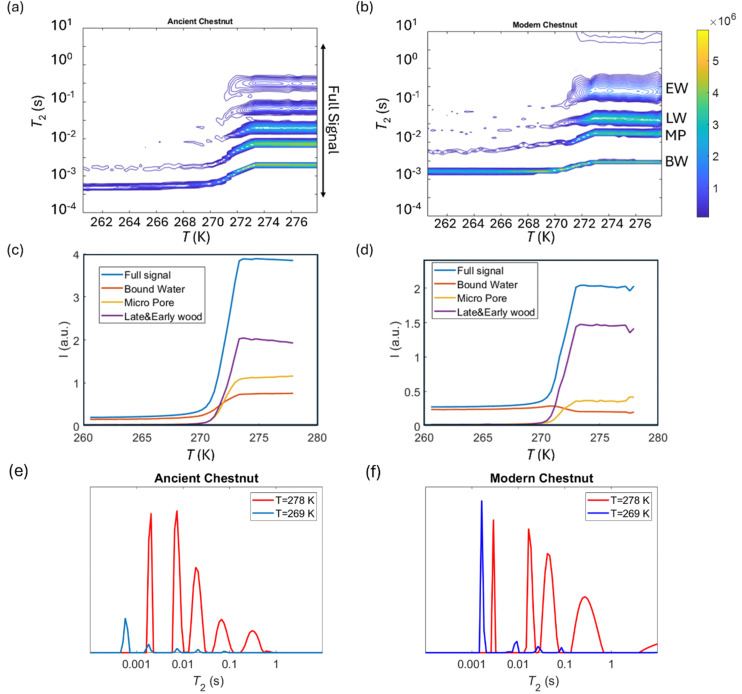
*T*_2_ relaxation distributions *versus* temperature for the ancient chestnut (a) and the modern chestnut (b). EW, LW, MP, and BW refer to earlywood, latewood, micropore and bound water signals, respectively. The signal intensity of each component *versus* temperature for the ancient chestnut (c) and the modern chestnut (d) and the full signal region indicated in (a). *T*_2_ distributions for the ancient chestnut (e) and the modern chestnut (f) before and after the melting point (*T* = 273 K).

**Fig. 10:**
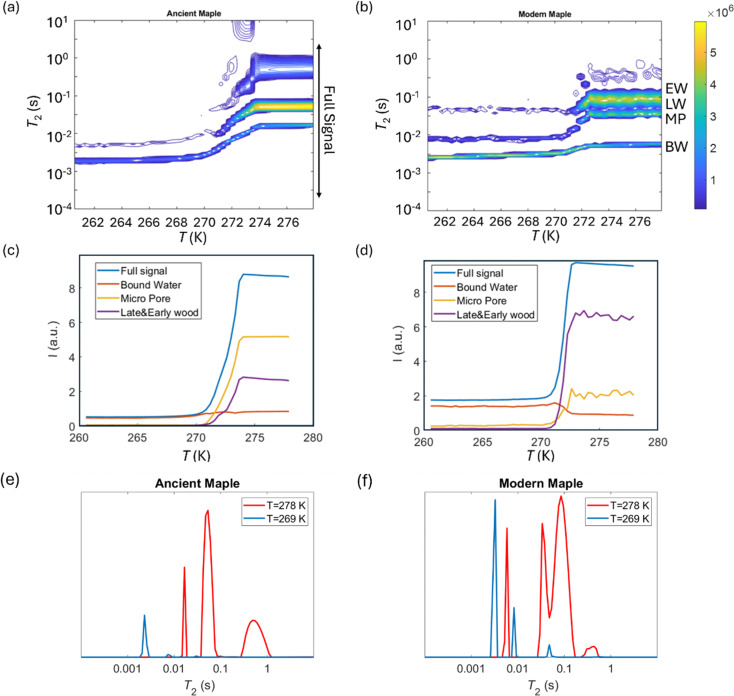
*T*_2_ relaxation distributions *versus* temperature for the ancient maple (a) and the modern maple (b). EW, LW, MP, and BW refer to earlywood, latewood, micropore and bound water signals, respectively. The signal intensity of each component *versus* temperature for the ancient maple (c) and the modern maple (d) and the full signal region indicated in (a). *T*_2_ distributions for the ancient maple (e) and the modern maple (f) before and after the melting point (*T* = 273 K).

*T*_2_ relaxation distributions and signal intensities are both plotted *versus* temperature for ancient and modern spruce, chestnut, and maple in Fig. 8–10, respectively. Moreover, as an example, the *T*_2_ distribution at two different temperatures, one above (*T* = 278 K) and one below (*T* = 269 K), and a melting point of 273 K are reported. Different signal contributions were separated, such as earlywood (EW), latewood (LW), micropore (MP) and bound water (BW). The two components observed below 273 K can be associated with two populations of bound water in the cell wall: BW has the shorter *T*_2_ and arises from water molecules hydrogen bonded to the hydroxyl groups and between the cellulose chains, whereas MP water has the longer *T*_2_ component and originates from water in cell wall micropores.^48,101^ It can be seen that, in all modern samples, the bound water signal intensity decreases after the bulk melting point, most probably because the bound water and free water signals are partially mixed due to molecular exchange.^48,102^ We note that the exchange cannot take place below the bulk melting point, as free water is frozen. A similar trend was not observed in the ancient counterparts, suggesting differences in the structure and dynamics. Furthermore, in all cases, water associated with tracheid/vessel lumens is frozen, as no clear signal is observed corresponding to those at lower temperatures, and as these structures are bigger in nature, the main melting point of these structures coincides with the bulk melting point of water (*T* = 273 K). In all the modern wood samples, the EW and LW signals constitute most of the full signal, while in the ancient counterparts, the relative amount of EW and LW water is significantly smaller, and other components (*i.e.*, cell walls) also play crucial role in the total signal, in good agreement with relaxometry results showing an increased *T*_2_ peak associated with water in cell walls. All ancient wood samples have MP water signals greater than that of their modern counterparts, indicating an increase of the micropore water content in the ancient wood samples. This result confirms the degradation suffered by the cell wall, corresponding to an increased permeability, which was already detected by relaxometry, diffusometry and micro-imaging. In the case of the ancient chestnut, also an extra signal is present (Fig. 9a), behaving similarly as the LW and EW signals, which is therefore likely attributed to similar bigger pore structures (*i.e.*, vessels) associated with the wood structure. This is in agreement with the increased compartmentalization of vessels in the ancient chestnut, characterized by three main relaxation components belonging to three different vessel sizes of around 260–350 μm, 170 μm and 80 μm detected from relaxation measurements and observed in μ-MR images.

**Fig. 11:**
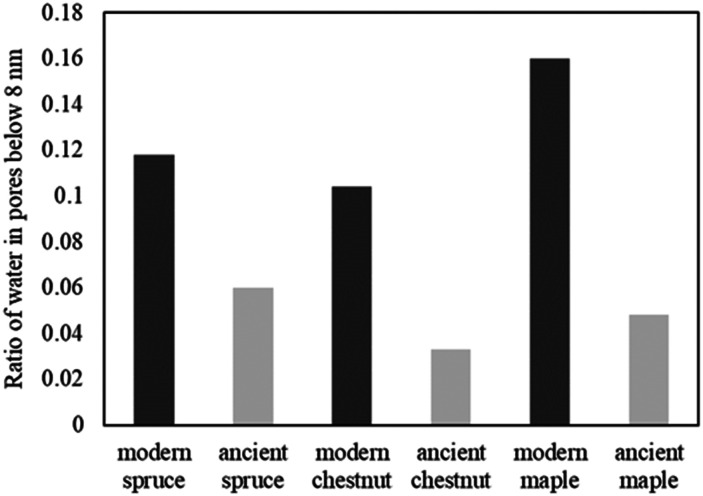
The amount of water in pores with a diameter of below 8 nm, corresponding to the ratio of the signal intensities measured at 265 K and above 273 K.

The relative amounts of water in pores with a size of below 8 nm, corresponding to the ratio of the signal intensities measured at 265 K and above 273 K, are shown in Fig. 11. The main result is that in all ancient wood samples it is smaller than in modern wood samples. The decrease of water portion in small pores corresponds to the decreased amount of bound water in the archaeological wood samples due to decay of cell wall structures.^30,103–105^

### Overall porosity

The combination of μ-MRI, relaxometry, diffusometry, and cryoporometry allowed us to cover several dimensional scales of wood samples, from cell wall cavities to sub-millimeter pores. In the following sections, we summarize the characteristics of the overall porosity and the different water distributions in each species of wood obtained by combining all the NMR techniques and we explain how these characteristics change as a result of the decay process.

### Spruce

Both the modern spruce and the ancient spruce are characterized by at least three water populations corresponding to three different pore sizes: earlywood tracheids, latewood tracheids and cell walls. In addition, the ancient spruce also shows a fourth population of water in the resin canals with a size of around 100 μm, which was not seen for the modern spruce where the resin canals, having a size of about 75 μm, are still full of resin rather than water. The earlywood tracheids in the ancient spruce have a larger diameter (∼36 μm) than in the modern spruce (∼32 μm). The similar *T*_1_ and *T*_2_ values along with the close diffusion coefficient (*D*_*x*2_) and pore size (*d*_2_) indicate that LW tracheids have similar sizes in both the modern spruce and the ancient spruce. The increase of *T*_2_ associated with water in cell walls and the decrease of the water amount in pores smaller than 8 nm indicate that the cell walls of the archaeological wood can retain less bound water. This is most probably due to the decay of the cell walls, and it may be possible that bigger pores appeared in the cell walls of the ancient wood samples. The decay process of the wood cell wall was also observed in the MR images in Fig. 1 and in the optical microscopy images reported in our previous work.^28^ Moreover, the non-isolated water compartments in EW tracheids, LW tracheids and cell walls, along with the increased signal of MP water, confirm the increase of the cell wall permeability due to its degradation. The higher tortuosity measured for the ancient spruce is in agreement with the greater number of components of the relaxation times *T*_1_ and *T*_2_ indicating the increase of water compartmentalization in the structure of the archaeological wood. This increased compartmentalization is due to the presence of inclusions, spores, and residues of fungal hyphae,^28^ and to the overall structural decay that led to the formation of new pores and water pools.

### Chestnut

The modern and the ancient chestnut belong to the hardwood group that is characterized by structures with different sizes that correspond to several water populations. The modern chestnut has three mean vessel sizes of around 300, 140, and 60 μm, whereas the ancient chestnut has four vessels sizes of about 350, 260, 170, and 80 μm. In the ancient chestnut, the vessels with sizes in the range of 260–350 μm are the most abundant. On average, the ancient chestnut is characterized by larger vessels compared to the modern chestnut and it shows a greater water compartmentalization, as indicated by its greater number of *T*_1_ and *T*_2_ components and by its higher mean tortuosity. The shortening of the *T*_1_ components in the ancient chestnut is caused by the significant presence of paramagnetic inclusions, *i.e.*, iron-tanning substances,^28,99^ in fibres, parenchyma and cell walls. Paramagnetic inclusions, that totally or partially obstruct vessels in the ancient sample, are also responsible for the formation of new compartments throughout the wood structure. Moreover, it is worth noting that, in the ancient wood, the *T*_2_ associated with water in vessels of size = 80 μm and with fibers/parenchyma is shorter than the *T*_2_ associated with water in vessels of size = 60 μm and fibers/parenchyma in the modern wood. This indicates that also the *T*_2_ may be shortened by the effect of paramagnetic substances. The decrease of the bound water amount in small pores (<8 nm) of the cell wall and the increased intensity of the MP water signal and of the *T*_1_ and *T*_2_ peaks associated with water in the cell walls of the ancient chestnut suggest a strong degradation process of the wood cell wall, which shows higher permeability and porosity, in good agreement with our previous work.^28^

### Maple

Three different water populations, corresponding to vessels, fibres and parenchyma, were detected for both the modern maple and the ancient maple. The modern maple is characterized by only one size of vessels, around 36 μm, whereas the ancient maple is characterized by two sizes, around 86 and 40 μm. Water is mostly stored in fibres and parenchyma, with a similar mean size of 25 μm in both the modern maple and the ancient maple. The similar size of fibres and parenchyma in both the wood samples is also confirmed by their close *T*_2_ values. The increase of *T*_2_ of water stored in cell walls in the archaeological maple suggests the increase of their size. Moreover, the decrease of the water content in pores below 8 nm of the cell walls along with the increase of the MP water signal in the ancient maple suggest a greater permeability of the cell walls. Again, the tortuosity is higher in the archaeological sample than in the modern sample, implying a more complex structure in the ancient wood. Indeed, the higher number of *T*_1_ and *T*_2_ components in the ancient sample indicates its greater structural compartmentalization that is induced by the decay process and the presence of spores and residues of fungal hyphae.^28^ However, the aforementioned results must be taken with care because the specimen comes from the tree trunk for the ancient maple and the specimen was collected from a branch for the modern maple. It may be that the total porosity and anatomical structures in the modern maple are not yet well developed compared to those in the ancient maple, due to their different growth stages.

## Conclusions

Research in the field of conservation sciences is constantly engaged in the search for innovative non-destructive techniques for the investigation of archaeological submerged wood. Ideal alternative techniques should allow obtaining information complementary or similar to that provided by conventional optical microscopy. This requirement has led to the advancement of the micro-imaging NMR technique with the optimization of its resolution such that it can resolve anatomical elements of wood with minimum dimensions around 10 micrometers. Additionally, wood anatomical features and water dynamics can also be characterized indirectly using techniques such as NMR relaxometry, NMR diffusometry, and NMR cryoporometry, which provide the characterization of both static and dynamic systems, *e.g.*, MR imaging *vs.* diffusion-weighted imaging (DWI). Specifically, NMR cryoporometry overcomes the resolution limit of MRI and diffusion and the paramagnetic bias of relaxometry, allowing us to go below 10 microns and investigate wood structures on the nanometric scale (*i.e.*, cell walls). Combining the above-mentioned NMR techniques and using appropriate mathematical models, it was possible to implement an innovative NMR protocol to obtain a comprehensive analysis of the porous wood structure. This work laid the foundation for the application of the new NMR protocol to the archaeological waterlogged wood. The multi-modal approach allowed us to detect the incipient cell wall decay, the accumulation of inclusions (black areas in MR images) and the biological infestations in the three archaeological wood samples studied in this work. Decay, inclusions, fungal spores, and hyphae have increased the structural complexity (corresponding to a higher tortuosity) of the ancient wood with the formation of new populations of water, identified by new *T*_1_ and *T*_2_ components, compared to the modern wood samples. All three ancient wood samples are characterized by a lower amount of water inside pores with a size of below 8 nm compared to the modern wood samples, indicating that the cell walls in the ancient wood hold more water in bigger pores and increase their permeability. To the best of our knowledge, this is the first study to jointly apply four different NMR techniques to encompass all the relevant length scales of wood. In future work, the protocol needs to be tested on a higher number of samples to verify its validity on several wood species. It would be preferable to develop two separate protocols, one for the characterization of softwood and the other for hardwood. Wood samples can have quite different structures depending on the individual, the age of the wood, and the location of the sample in the trunk/branch. Therefore, the differences between modern and ancient wood samples will require careful evaluation and correct interpretation.

## Data availability

Data for this article are available at “STAGNO 2024” at 10.6084/m9.figshare.26196911.

## Conflicts of interest

There are no conflicts to declare.
